# Ketone Body 3‐Hydroxybutyrate Ameliorates Atherosclerosis via Receptor Gpr109a‐Mediated Calcium Influx

**DOI:** 10.1002/advs.202003410

**Published:** 2021-03-01

**Authors:** Shu‐jie Zhang, Zi‐hua Li, Yu‐dian Zhang, Jin Chen, Yuan Li, Fu‐qing Wu, Wei Wang, Zong Jie Cui, Guo‐Qiang Chen

**Affiliations:** ^1^ School of Life Sciences Tsinghua University Beijing 100084 P. R. China; ^2^ Institute of Cell Biology Beijing Normal University Beijing 100875 P. R. China; ^3^ Innovative Institute of Animal Healthy Breeding College of Animal Sciences and Technology Zhongkai University of Agriculture and Engineering Guangzhou 510025 P. R. China; ^4^ Key Laboratory of Zoonosis Research Ministry of Education College of Veterinary Medicine Jilin University Changchun 130062 P. R. China; ^5^ Tsinghua‐Peking Center for Life Sciences Tsinghua University Beijing 100084 P. R. China; ^6^ Center for Synthetic and Systems Biology Tsinghua University Beijing 100084 P. R. China; ^7^ MOE Key Laboratory for Industrial Biocatalysis Dept Chemical Engineering Tsinghua University Beijing 100084 P. R. China

**Keywords:** 3‐HB, atherosclerosis, calcium influx, cholesterol efflux, M1 macrophages, NLRP3, PHB

## Abstract

Atherosclerosis is a chronic inflammatory disease that can cause acute cardiovascular events. Activation of the NOD‐like receptor family, pyrin domain containing protein 3 (NLRP3) inflammasome enhances atherogenesis, which links lipid metabolism to sterile inflammation. This study examines the impact of an endogenous metabolite, namely ketone body 3‐hydroxybutyrate (3‐HB), on a mouse model of atherosclerosis. It is found that daily oral administration of 3‐HB can significantly ameliorate atherosclerosis. Mechanistically, 3‐HB is found to reduce the M1 macrophage proportion and promote cholesterol efflux by acting on macrophages through its receptor G‐protein‐coupled receptor 109a (Gpr109a). 3‐HB–Gpr109a signaling promotes extracellular calcium (Ca^2+^) influx. The elevation of intracellular Ca^2+^ level reduces the release of Ca^2+^ from the endothelium reticulum (ER) to mitochondria, thus inhibits ER stress triggered by ER Ca^2+^ store depletion. As NLRP3 inflammasome can be activated by ER stress, 3‐HB can inhibit the activation of NLRP3 inflammasome, which triggers the increase of M1 macrophage proportion and the inhibition of cholesterol efflux. It is concluded that daily nutritional supplementation of 3‐HB attenuates atherosclerosis in mice.

## Graphical abstract

This study demonstrates that daily oral administration of ketone body 3‐hydroxybutyrate (3‐HB) attenuates atherosclerosis. Mechanistically, 3‐HB reduces M1 macrophage proportion and promotes cholesterol efflux by acting on macrophages through the Gpr109a–NLRP3 pathway. 3‐HB inhibits ER calcium deletion induced NLRP3 inflammasome activation via 3‐HB–Gpr109a signaling‐mediated extracellular Ca^2+^ influx in macrophages.

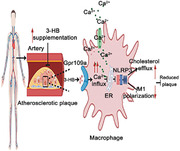

## 1 Introduction

Atherosclerosis is by far the most common cardiovascular disease although not fatal in most cases.^[^ ^1^ ^]^ However, plaque ruptures and thrombosis can cause acute cardiovascular events, such as stroke, heart attack, and some other serious cases.^[^ ^2^ ^]^ Atherosclerosis is characterized by the accumulation of lipid‐loaded macrophages in the arterial wall, which is attributed to the disorder of lipid metabolism and adaptive immune response.^[^ ^3^ ^]^ Dynamically changes of macrophages play a decisive role in the initiation and progression of atherosclerosis.^[^ ^4^ ^]^ Both the number and the inflammatory phenotype of macrophages determine the progression of plaques.^[^ ^5^ ^]^

Macrophages in the plaque have been continuously exposed to various forms of lipids and oxidized lipoproteins leading to activation of inflammatory genes and increasing the proportion of proinflammatory M1 macrophages.^[^ ^6^ ^]^ In an inflammatory microenvironment, NOD‐like receptor family, pyrin domain containing protein 3 (NLRP3) inflammasome is activated and therefore promotes M1 macrophage differentiation.^[^ ^7^, ^8^ ^]^

NLRP3 inflammasome is a pivotal therapeutic target in atherosclerosis.^[^ ^9^ ^]^ NLRP3 can be activated by diverse stimuli signals in macrophages such as ER stress, reactive oxygen species (ROS), and excessive calcium (Ca^2+^). Interleukin‐1*β* (IL‐1*β*) and IL‐18, which are the mature products of NLRP3 inflammasome activation, have proatherogenic and proinflammatory roles in atherosclerosis.^[^ ^10^ ^]^ They are highly expressed by macrophages in *apoE* ^−/−^ mice.^[^ ^11^ ^]^ Deletion of *IL‐1β* gene in *apoE* ^−/−^ mice reduced the plaque area compared with the controls.^[^ ^12^ ^]^ Reduced IL‐1*β* level in *apoE* ^−/−^ mice is accompanied with decreased levels of inflammatory cytokines including tumor necrosis factor (TNF‐*α*), interleukin 6 (IL‐6), intercellular cell adhesion molecule‐1 (ICAM‐1), and vascular cell adhesion molecule‐1 (VCAM‐1).^[^ ^13^ ^]^ IL‐1*β* can also induce a negative feedback, which inhibits Gpr109a–ABCA1 (ATP‐binding cassette subfamily A1), and therefore leads to the accumulation of cholesterol in macrophages and the formation of foam cells.^[^ ^14^ ^]^

Gpr109a is a G‐protein coupled receptor (GPCRs) expressed on adipocytes, macrophages, and neutrophils.^[^ ^15^ ^]^ Activation of Gpr109a by niacin (NA) reduces progression of atherosclerosis via exerting a lipid lowering effect as well as a direct anti‐inflammatory property.^[^ ^16^, ^17^ ^]^

3‐Hydroxybutyrate (3‐HB) is produced by fatty acid *β*‐oxidation in the liver and transported to the extrahepatic tissues such as heart, brain, and muscle to serve as an energy source.^[^ ^18^ ^]^ 3‐HB can be available from microbial poly‐(3‐hydroxybutyrate),^[^ ^19^ ^]^ which has important applications in the field of nutrition and medicine.^[^ ^20^ ^]^ 3‐HB has been reported to have diverse cell signaling and protective functions; it directly promotes 3‐hydroxybutyrylation of some proteins and functions as an endogenous inhibitor of histone deacetylases as well as an agonist of Gpr109a.^[^ ^21^ ^]^ Previous studies showed that 3‐HB has various potential benefits for cardiovascular disease treatments.^[^ ^22^ ^]^ It was reported that elevation of circulating 3‐HB level showed an obvious improvement in patients with chronic heart failure.^[^ ^23^ ^]^ But these are not necessarily directly impacting atherosclerosis.

Above all, these striking researches raise some questions on 3‐HB: whether exogenous supplement of 3‐HB has the beneficial effect of relieving atherosclerosis? If so, is there a different mechanism underlying the beneficial effect of 3‐HB?

To date, various GPCRs have been identified to participate in the activation and repression of NLRP3 inflammasome by sensing their agonists.^[^ ^24^ ^]^ Substantially increased extracellular Ca^2+^ level can activate the NLRP3 inflammasome via GPRC6A.^[^ ^25^ ^]^ It was demonstrated that Gpr109a and Gpr43 acting as short‐chain fatty acid (SCFA) receptors can also activate NLRP3 inflammasome in intestinal epithelial cells via K^+^ efflux‐mediated Ca^2+^ immobilization.^[^ ^26^ ^]^ Activation of Gpr109a can protect intestinal epithelial cells from apoptosis; however, activation of NLRP3 inflammasome can exacerbate intestinal epithelial cells’ damage,^[^ ^27^ ^]^ and these results are contradictory. Thus, there may be a new association among 3‐HB–Gpr109a signaling, Ca^2+^ immobilization, and NLRP3 inflammasome activation.

This study aimed to investigate if intragastric administration of 3‐HB could attenuate atherosclerosis in mouse models and its related mechanism.

## 2 Results

### 2.1 3‐HB Treatment Reduces Systemic Inflammatory Response in Atherosclerosis Mice

**Figure 1:**
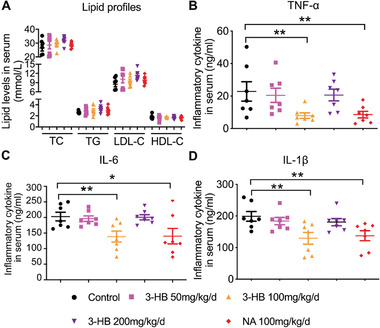
Effects of 3‐HB on plasma lipid profiles and inflammatory cytokines levels in mice, respectively. Five week old male *apoE* ^−/−^ mice were fed with a 1.25% high‐cholesterol diet for 9 weeks and concomitantly treated daily with 3‐HB (50, 100, and 200 mg kg^−1^ d^−1^) and NA (100 mg kg^−1^ d^−1^) for 9 weeks via intragastric administration, respectively (*n* = 7–9 per group). A) Changes of lipid profiles in the mouse serum (*n* = 7 per group). TC: total cholesterol; TG: total triglyceride; LDL‐C: low‐density lipoprotein cholesterol; HDL‐C: high‐density lipoprotein cholesterol. B–D) Plasma TNF‐*α*, IL‐6, and 1L‐1*β*concentrations (*n* = 7 per group). IL‐6: interleukin 6; TNF‐*α*: tumor necrosis factor; IL‐1*β*: interleukin‐1*β*. Data are presented as the mean±SEM from at least three independent experiments, one‐way ANOVA, * *p* < 0.05, ** *p* < 0.01.

*ApoE* ^−/−^ mice can spontaneously develop atherosclerosis; thus, they are commonly used as the animal model for atherosclerosis research. A cholesterol‐rich diet can aggravate atherosclerosis in *apoE* ^−/−^ mice.^[^ ^28^ ^]^ To evaluate the efficacy of 3‐HB treatment for atherogenesis, *apoE* ^−/−^ mice were fed a cholesterol‐rich diet and concomitantly administrated with 3‐HB, NA (positive control), and 0.9% saline (vehicle control) intragastrically for 9 weeks, respectively. Results showed that 3‐HB significantly decreased the body weights of *apoE* ^−/−^ mice fed with a high‐fat diet compared to the negative control group (Figure S1, Supporting Information). However, no difference was observed in the plasma lipid profiles between mice with and without 3‐HB, indicating that 3‐HB did not have a significant impact on the overall lipid metabolism (**Figure** **1**A). As chronic inflammation is a significant feature of atherosclerosis, we next examined the levels of inflammatory factors. Our results showed that the levels of proinflammatory cytokines TNF‐*α*, IL‐6, and IL‐1*β* were suppressed by 3‐HB treatment (Figure 1B–D). Among the three 3‐HB concentrations, the dose of 3‐HB (100 mg kg^−1^ d^−1^) generated the best response (Figure 1B–D). These data demonstrated that 3‐HB treatment significantly reduced the systemic inflammatory response in atherosclerosis model mice.

### 2.2 3‐HB Treatment Reduces Atherogenesis

**Figure 2:**
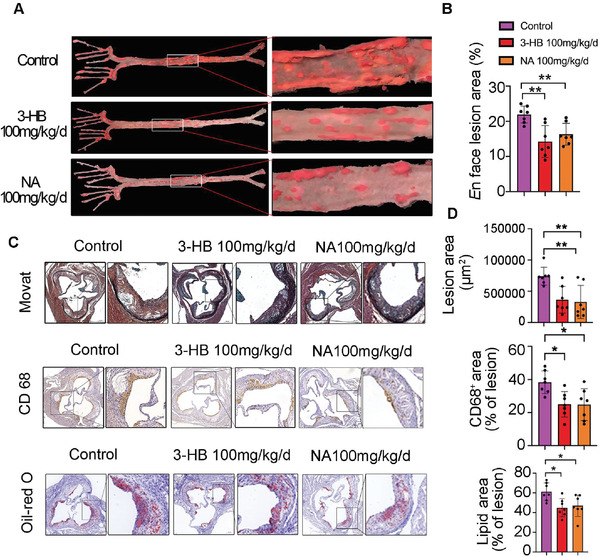
3‐HB treatment reduces murine atherogenesis. A,B) Representative images of en face Oil Red O stained aortas and quantification of aortic plaque burden of mice treated with or without 3‐HB (100 mg kg^−1^ d^−1^) and NA (100 mg kg^−1^ d^−1^), respectively (*n* = 7 per group). C,D) Representative images and quantification of aortic root lesion areas and plaque compositions. Movat staining measurement of aortic lesion areas (*n* = 7); CD68 immuno‐histochemical staining measurement of macrophage content (*n* = 7); Oil Red O staining measurement of lipid content (*n* = 7). Scale bar = 200 µm. Data were presented as the mean±SEM from at least three independent experiments, one‐way ANOVA, * *p* < 0.05, and ** *p* < 0.01.

To further study the effect of 3‐HB on atherosclerosis in the model mice, the aortas were collected from the 3‐HB (100 mg kg^−1^ d^−1^), niacin (100 mg kg^−1^ d^−1^), and 0.9% saline (vehicle control) treated groups, respectively. The atherosclerotic plaques in the whole aortas were stained with Oil Red O. It was clearly observed that overall plaque burden in the entire aortas of 3‐HB‐treated mice was remarkably reduced compared with the untreated control group (**Figure** **2**A,B). 3‐HB showed almost an identical effect as niacin (positive control) (Figure 2A,B). The lesion areas, intraplaque macrophage infiltration, and lipid deposition in the plaque of the aortic roots were decreased in 3‐HB effective‐dose‐treated group compared to the control group (Figure 2C,D). However, contents of smooth muscle cells in the plaques were not detectably affected by 3‐HB (Figure S2, Supporting Information). These data demonstrate that 3‐HB treatment is effective in reducing the atherogenesis. It becomes very interesting to investigate the mechanism of 3‐HB on atherosclerosis.

### 2.3 3‐HB Mediates Inflammatory Response and Cholesterol Efflux in *apoE* ^−/−^ Mice

**Figure 3:**
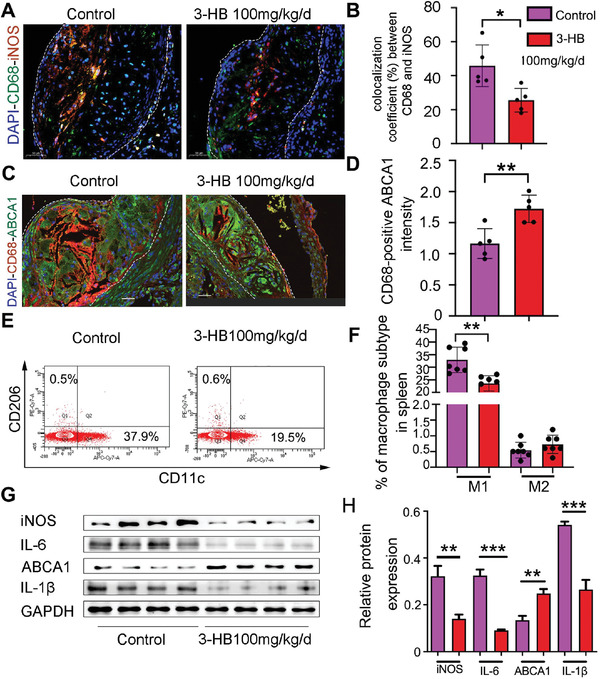
3‐HB reduces M1 macrophages and promotes cholesterol efflux in *apoE* ^−/−^ mice. A,B) Immunofluorescence staining for iNOS (red) and CD68 (green) in the aortic root sections and quantification of colocalization, percentage of iNOS^+^ area in CD68^+^ area, respectively. DAPI was stained for cell nucleus (blue), Scale bar = 50 µm (*n* = 5 per group). C,D) ABCA1 expression (green) in macrophages (red) was assessed by immunofluorescence staining and quantification of ABCA1 fluorescence intensity overlapping with CD68^+^ area, respectively. Scale bar = 30 µm (*n* = 5 per group). E,F) Western blot analysis of inflammatory markers (IL‐6, iNOS, and IL‐1*β*) and cholesterol efflux marker (ABCA1) in the aortas from mice treated with or without 3‐HB and quantification of protein expression, respectively (*n* = 4 per group). G,H) Flow cytometry plots and quantitative analysis of macrophage differentiation in the spleen of *apoE* ^−/−^ mice treated with or without 3‐HB (100 mg kg^−1^ d^−1^) (*n* = 7 per group), respectively. Data are presented as the mean±SEM from at least three independent experiments, Student's *t*‐test, * *p* < 0.05, ** *p* < 0.01, and *** *p* < 0.001.

As reported, macrophages that infiltrated into plaques often play a crucial role in the development of atherosclerosis.^[^ ^29^ ^]^ Intraplaque proinflammatory microenvironment can switch the macrophages in the plaques to M1 macrophages, which play a major role in proinflammatory factors’ secretion.^[^ ^30^ ^]^ Modulation of macrophage phenotypes is a novel strategy for the treatment of atherosclerosis.^[^ ^31^ ^]^ Since 3‐HB reduced intraplaque macrophage infiltration of *apoE* ^−/−^ mice (Figure 2), we set out to determine whether 3‐HB can reduce the proportion of intraplaque M1 macrophages. We observed that M1 macrophage marker inducible nitric oxide synthase (iNOS) decreasingly co‐localized with CD68^+^ cells (macrophages) in the aortic sinus sections of 3‐HB‐treated mice after immunofluorescent analysis (**Figure** **3**A,B). These results were consistent with the flow cytometric analysis of M1 macrophage proportion in the spleen of mice treated with 3‐HB (100 mg kg^−1^ d^−1^) (Figure 3E,F).

Proinflammatory M1 macrophages can phagocytiose excess cholesterol and inhibit cholesterol efflux leading to the formation of foam cells, which contribute to atherosclerotic plaque growth.^[^ ^32^ ^]^ ATP‐binding cassette transporter A1 (ABCA1) and ATP‐binding cassette transporter G1 (ABCG1) are crucial transporters for cholesterol efflux.^[^ ^33^, ^34^ ^]^ To investigate if 3‐HB reduced the lipid deposition in plaques attributing to its promotion of cholesterol efflux in macrophages, we next investigated the co‐localization of ABCA1 and CD68 in the aortic root sections of *apoE* ^−/−^ mice. Immunofluorescent staining data clearly showed that 3‐HB significantly increased ABCA1 expression (labeled with the green dye) in the CD68^+^ macrophages (red) in the plaques (Figure 3C,D).

Consistent with the above results (Figure 3A–F), 3‐HB was found to suppress the inflammatory markers of M1 macrophages (IL‐6 and iNOS), promote cholesterol efflux transporter ABCA1, and decrease 1L‐1*β* expression at protein level in the aortas of 3‐HB‐treated mice (Figure 3G,H). Since 1L‐1*β* is a key link between the innate inflammation and cholesterol metabolism, we speculated that 3‐HB may reduce intraplaque M1 macrophage infiltration and promote cholesterol efflux dependent on the inhibition of NLRP3 inflammasome.

### 2.4 3‐HB Suppresses M1 Polarization and Promotes Cholesterol Efflux via Gpr109a‐Mediated Inhibition of NLPR3 Inflammasome in Macrophages

**Figure 4:**
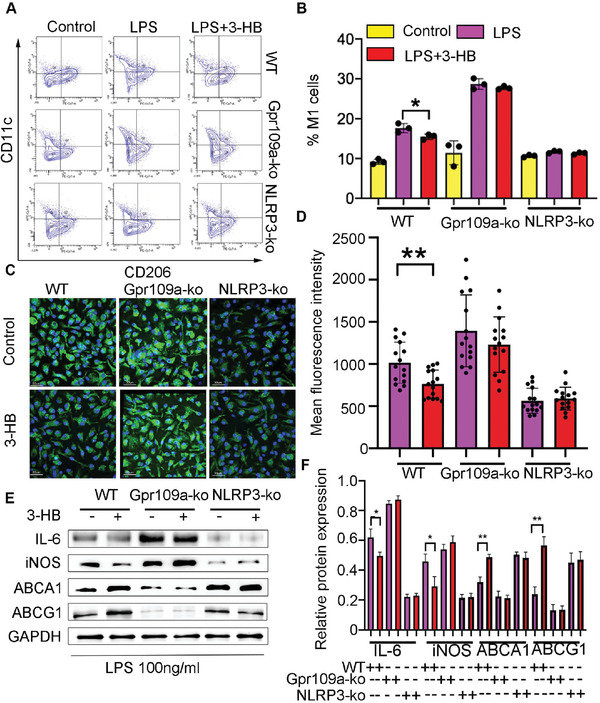
3‐HB reduces M1 macrophages and promotes cholesterol efflux in the presence of Gpr109a which activation inhibits NLRP3 in BMDMs. A,B) Percentages of M1 subsets in LPS (100 × 10^−9^ g mL^−1^) treated BMDMs from *WT*, *Gpr109a* ^−/^ *^−^*, and *NLRP3* ^−/−^ mice treated with or without 3‐HB (10 × 10^−3^ m), respectively. BMDMs were incubated with LPS (100 × 10^−9^ g mL^−1^) for 24 h, simultaneously added 3‐HB (10 × 10^−3^ m) and then analyzed by flow cytometry (*n* = 3). Data were expressed as mean±SEM from at least three independent experiments, one‐way ANOVA, * *p* < 0.05. C,D) Representative images of NBD‐cholesterol‐loaded BMDMs obtained by confocal laser reflection microscopy and quantification of the NBD (green) fluorescence in BMDMs, respectively (*n* = 15). Scale bar = 30 µm. BMDMs from *WT*, *Gpr109a* ^−/−^, *NLRP3* ^−/−^ mice were incubated with or without 3‐HB (10 × 10^−3^ m) for 24 h. Data were expressed as mean±SEM from at least three independent experiments, Student's *t*‐test, * *p* < 0.05. E,F) Typical pictures of M1 markers (iNOS and IL‐6) and cholesterol efflux markers (ABCA1 and ABCG1) expressed in protein level analyzed by western blot and the stripes were statistically analyzed by Image J software, respectively (*n* = 3). Data were expressed as mean±SEM from at least three independent experiments, one‐way ANOVA, * *p* < 0.05, ** *p* < 0.01.

As described in Figure 3, 3‐HB has been shown to inhibit M1 polarization and promote cholesterol efflux in vivo. Further studies were conducted to investigate its underlying molecular mechanism. 3‐HB has been reported to activate its endogenous receptor Gpr109a on cell membranes among various cell signaling functions.^[^ ^35^ ^]^ Given that 3‐HB was quickly metabolized in vivo and that 3‐HB generated faster outcomes through Gpr109a,^[^ ^36^ ^]^ it is likely that 3‐HB exerted its protective role in atherosclerosis through activating Gpr109a signaling rather than though other mechanisms requiring chromatin remodeling, especially when Gpr109a is highly expressed in macrophages.^[^ ^37^ ^]^ 3‐HB effectively reduces IL‐1*β* (the product of NLRP3 inflammsome activation) secretion (Figure 1). Since IL‐1*β* links to cholesterol and innate immune responses, we speculated that 3‐HB exerts its atheroprotection by activating its receptor on macrophages thereby inhibiting NLRP3 activation. To verify this hypothesis, we isolated Bone‐marrow‐derived macrophages (BMDMs) were isolated from wild‐type (*WT*) mice, *Gpr109a* ^−/−^ mice, and *NLRP3* ^−/−^ mice, respectively, and incubated with lipopolysaccharide (LPS, 100 × 10^−9^ g mL^−1^) for 24 h to induce M1 polarization with or without 3‐HB (10 ×10 ^−3^ m). As expected, 3‐HB inhibited the formation of the M1 macrophages (CD11c^+^ subtype cells) primed by LPS, whereas it was undetectable in *Gpr109a* ^−/−^ BMDMs (**Figure** **4**A,B). These data show that 3‐HB impedes macrophage M1 polarization via its receptor Gpr109a. In addition, we observed no differences in *NLRP3* ^−/−^ BMDMs treated with or without 3‐HB (Figure 4A,B). These results suggest that 3‐HB inhibits M1 polarization via Gpr109a‐mediated inhibition of NLRP3 inflammasome.

Intraplaque macrophages take up excessive cholesterol and transform into unstable foam cells; a process impairs macrophages’ function and promotes atherogenesis.^[^ ^38^ ^]^ This process can be mimicked in vitro by incubating macrophages with 22‐(*N*‐(7‐nitrobenz‐2‐oxa‐1,3‐diazol‐4‐yl)amino)‐23,24‐bisnor‐5‐cholen‐*β*‐Ol (NBD)‐labeled cholesterol followed by visualizing with a confocal microscope.^[^ ^39^ ^]^ To study how 3‐HB affects the capacity of macrophage cholesterol efflux, the *WT*, *NLRP3* ^−/−^, and Gpr109a^‐/‐^ BMDMs were treated with or without 3‐HB (10 ×10 ^−3^ m) for 24 h, respectively. Subsequently, these treated macrophages were added with NBD‐cholesterol, then the NBD signals were detected using a confocal microscope. Results showed that 3‐HB increased the cholesterol efflux capacity of BMDMs from *WT* mice, whereas lost this ability in both the *NLRP3* ^−/^ *^−^* and the *Gpr109a* ^−/−^ BMDMs (Figure 4C,D).

Finally, we confirmed the above results at protein level by using western blot analysis. In agreement with above results, 3‐HB decreased the expression of M1 macrophage markers including IL‐6/iNOS and increased ABCA1/ABCG1 expression (Figure 4E,F). Also, the effects of 3‐HB disappeared in both *Gpr109a* ^−/−^ and *NLRP3* ^−/−^ BMDM cells (Figure 4E,F). Taken together, these results suggest that 3‐HB inhibits M1 polarization while promoting cholesterol efflux in macrophages by inhibiting NLRP3 inflammasome via the Gpr109a receptor.

### 2.5 3‐HB Stabilizes ER Ca^2+^ Storage through 3‐HB‐Gpr109a Signaling‐Induced Calcium Influx

**Figure 5:**
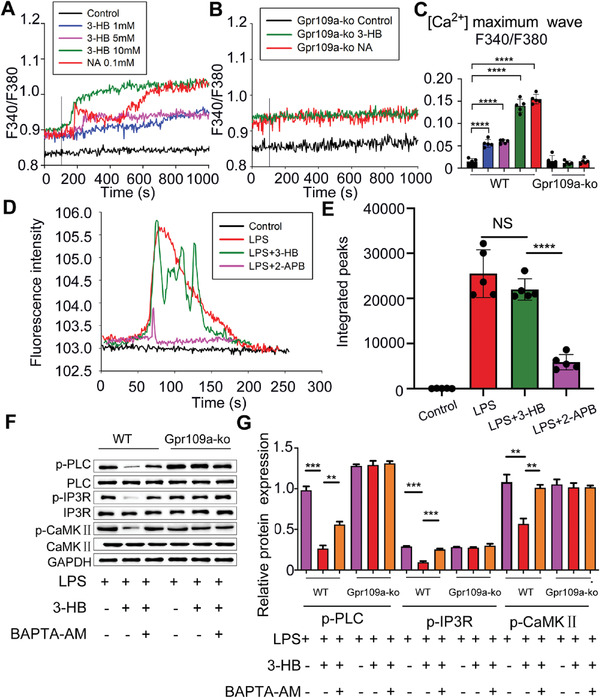
3‐HB inhibits Ca^2+^ releasing from ER depended on 3‐HB–Gpr109a signaling‐activated Ca^2+^ influx. A,B) Change in cytosolic Ca^2+^ concentration in real time as detected by Fura‐2 fluorescence imaging. The isolated BMDMs from *WT* or *Gpr109a* ^−/−^ mice were loaded with Fura‐2 AM (10 × 10^−6^ m), and then perfused in Hank's buffer (with added Ca^2+^). 3‐HB (1 × 10^−3^, 5 × 10^−3^, and 10 × 10^−3^ m) or niacin (0.1 × 10^−6^ m) were added as indicated by the vertical lines in the Ca^2+^ tracings. C) Increases in intracellular Ca^2+^ concentration after the addition of 3‐HB (1 × 10^−3^, 5 ×10^−3^, and 10 × 10^−3^ m) or niacin (0.1 × 10^−6^ m) were calculated and plotted (*n* = 5). D) Change in cytosolic Ca^2+^ concentration in real time detected via Fluo‐4 fluorescence imaging. The isolated BMDMs from *WT* mice were loaded with Fluo‐4 AM (10 × 10^−6^ m), and then perfused in Hank's buffer (without addition of Ca^2+^). LPS (100 × 10^−9^ g mL^−1^), LPS (100 × 10^−9^ g mL^−1^) +3‐HB (10 × 10^−3^ m) or LPS (100 × 10^−9^ g mL^−1^) +2‐APB (100 × 10^−6^ m) were added as indicated using Ca^2+^ tracking. E) Increases in intracellular Ca^2+^ concentration after the addition of LPS (100 × 10^−9^ g mL^−1^) +3‐HB (10 × 10^−3^ m) or LPS (100 × 10^−9^ g mL^−1^) +2‐APB (100 × 10^−6^ m) (*n* = 5). F,G) Changes of phosphorylation levels of protein PLC, IP3R, and CaMKII in BMDMs treated with LPS (100 × 10^−9^ g mL^−1^) with or without 3‐HB (10 × 10^−3^ m) or the intracellular calcium chelater BAPTA‐AM (20 × 10^−6^ m). Data are presented as mean±SEM from at least three independent experiments, one‐way ANOVA. ** *p* <0.01, *** *p* <0.01, **** *p* <0.01; NS, no statistical significance.

We next assessed the impact of 3‐HB signaling on NLRP3 inflammasome activity in BMDMs. Ca^2+^ is a critical second messenger that involves in nearly every aspect of cellular physiological and pathophysiological processes, including innate immune response.^[^ ^40^ ^]^ We next investigated the influence of 3‐HB–Gpr109a signaling on cytosolic calcium concentration. BMDM cells were loaded with the fluorescent Ca^2+^ indicator Fura‐2 acetoxymethyl ester (AM). The Fura‐2 AM‐loaded BMDM cells were then perfused with Hank's buffer (with added Ca^2+^), followed by the additions of various concentrations of 3‐HB and the positive control niacin, respectively. Ca^2+^ signals in each cell detected in real time showed that 3‐HB induced sustained intracellular Ca^2+^ accumulation in a 3‐HB dose‐dependent manner almost on the same level as the positive control niacin (**Figure** **5**A,C). The activation of Ca^2+^ influx by 3‐HB and niacin was not observed in the *Gpr109a^−^* ^/−^ BMDMs (Figure 5B,C), suggesting that 3‐HB promotes the calcium influx by activation of Gpr109a. 3‐HB did not elevate intracellular Ca^2+^ level in the absence of extracellular Ca^2+^, demonstrating that Ca^2+^ level elevated by 3‐HB should be sourced from extracellular environments (Figure S3, Supporting Information).

ER is the major intracellular calcium storage and responsible for the regulation of cytoplasmic calcium homeostasis.^[^ ^41^ ^]^ We next investigated how 3‐HB–Gpr109a signaling‐mediated calcium influx influence ER Ca^2+^ homeostasis. BMDM cells were incubated with fluorescent Ca^2+^ indicator Fluo‐4 AM. The Fluo‐4 AM‐loaded BMDM cells were then perfused with Hank's buffer (without added Ca^2+^), followed by the addition of LPS (100 × 10^−9^ g mL^−1^), LPS (100 × 10^−9^ g mL^−1^) + 3‐HB (10 × 10^−3^ m) or LPS (100 × 10^−9^ g mL^−1^) + 2‐Aminoethoxydiphenyl borate (2‐APB) (100 × 10^−6^ m), respectively. As can be seen, LPS could elevate calcium levels in cells, and in the presence of 2‐APB, which is an inhibitor of IP3R calcium channel, the cytoplasmic calcium ions’ concentration elevated by LPS was significantly reduced. These data suggested that LPS could promote calcium ions releasing from ER. Without extracellular calcium ions, 3‐HB could not inhibit the release of calcium ions from ER primed by LPS (Figure 5D,E). It is reported that PLC–IP3R–CaMKII cell signaling pathway is responsible for regulating ER calcium homeostasis.^[^ ^42^ ^]^ Since 3‐HB showed an inhibitory effect in ER calcium ions’ release, 3‐HB was expected to inhibit the activation of phospholipase C (PLC)–inositol 1,4,5‐trisphosphate (IP3R)–calmodulin‐dependent protein kinases II (CaMKII) signaling pathway. Therefore, the changes of PLC–IP3R–CaMKII signaling pathway were studied in cells treated with or without 3‐HB (10 × 10^−3^ m) by western blot analysis. Results showed that 3‐HB inhibited the activation of PLC–IP3R–CaMKII signaling pathway in *WT* BMDMs, and the intracellular Ca^2+^ chelator 1,2‐bis[2‐aminophenoxy]ethane‐*N,N,N′,N′*‐tetraacetic acid tetrakis [acetoxymethyl ester] (BAPTA‐AM) abolished 3‐HB‐mediated inhibition. As expected, 3‐HB combined with or without BAPTA‐AM had no impact on PLC–IP3R–CaMKII pathway in *Gpr109a* ^−/−^ BMDMs (Figure 5F,G). It can be concluded that Ca^2+^ influx induced by 3‐HB–Gpr109a signaling inhibits PLC–IP3R–CaMKII cell signaling pathway and therefore stabilizes Ca^2+^ ER storage.

### 2.6 3‐HB–Gpr109a Signaling Inhibits NLRP3 Inflammasome Activation

**Figure 6:**
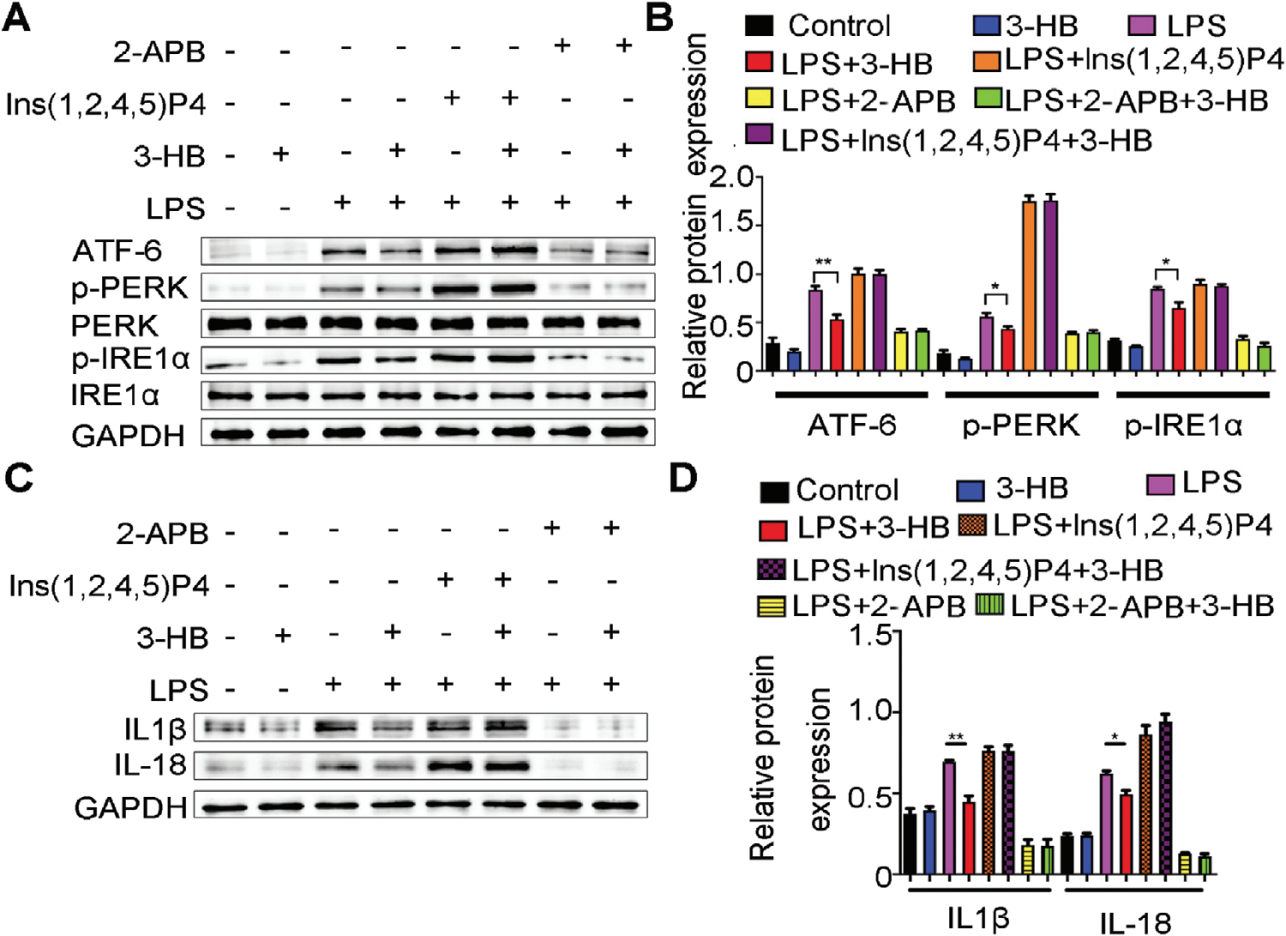
3‐HB inhibits ER stress‐induced activation of NLRP3 inflammasome via stabilizing ER Ca^2+^ storage. A,B) Western blot analysis of ER stress sensors (PERK, ATF‐6, and IRE1*α*) changes of BMDMs’ cell lysate. BMDMs were stimulated with LPS (100 × 10^−9^ g mL^−1^) and then added with the IP3R inhibitor 2‐APB (100 × 10^−6^ m)/IP3R agonist Ins(1,2,4,5)P4) (1 × 10^−6^ m) with or without 3‐HB (10 × 10^−3^ m). C,D) 3‐HB inhibits ER stress‐induced NLPR3 inflammasome activation. BMDMs were stimulated with LPS (100 × 10^−9^ g mL^−1^) to mimic the microenvironment of plaque, and meanwhile co‐incubated with IP3R inhibitor 2‐APB (100 × 10^−6^ m)/ IP3R agonist Ins(1,2,4,5)P4) (1 × 10^−6^ m) with or without 3‐HB (10 × 10^−3^ m), and then analysis of the mature products (IL‐18 and IL‐1*β*) of NLRP3 inflammasome activation. The data are presented as mean±SEM from at least three independent experiments and analyzed by the one‐way ANOVA, * *p*<0.05, ** *p*<0.01.

ER Ca^2+^ storage depletion has been reported to induce ER stress. and protein kinase RNA‐like endoplasmic reticulum kinase (PERK), activating transcription factor‐6 (ATF6), and inositol‐requiring enzyme‐1 α (IRE1*α)* are three ER stress sensors.^[^ ^43^ ^]^ 3‐HB suppressed ER stress in LPS‐stimulated BMDMs; however, with the addition of Ins(1,2,4,5)P4 (an agonist of IP3R) or 2‐APB (an inhibitor of IP3R), 3‐HB failed to ameliorate ER stress (**Figure** **6**A,B). These results revealed that 3‐HB–Gpr109a signaling attenuated ER stress induced by ER Ca^2+^ storage depletion through inhibiting the phosphorylation of IP3R.

It is reported that ER stress is required to activate NLRP3 inflammasome in macrophages.^[^ ^44^ ^]^ To further answer the question whether 3‐HB affects the activation of NLRP3 inflammasome via inhibiting the release of calcium from ER, we primed BMDMs with LPS and stimulated them with 3‐HB, and we found that 3‐HB lowered inflammatory cytokine IL‐18 and IL‐1*β* expressions in BMDMs (Figure 6C,D). In the presence of Ins(1,2,4,5)P4 or 2‐APB, 3‐HB lost its inhititory function of IL‐18 and IL‐1*β*.^[^ ^8^ ^]^ These results here further indicated that 3‐HB–Gpr109a signaling inhibited NLRP3 inflammasome activation through stabilizing ER calcium storage.

### 2.7 3‐HB Attenuates Atherosclerosis via Gpr109a Expressed on Bone‐Marrow‐Derived Macrophages

**Figure 7:**
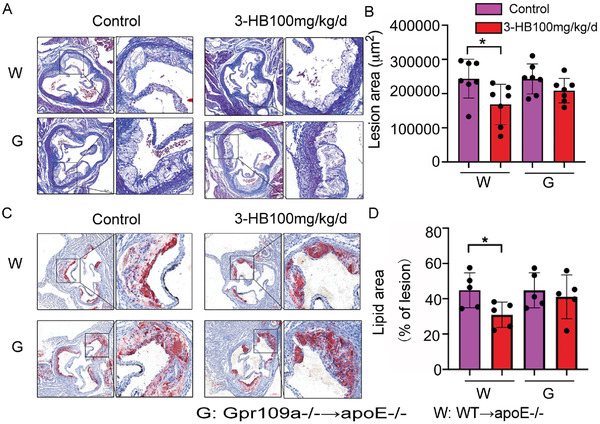
3‐HB attenuates atherosclerosis dependent on the Gpr109a expressing bone‐marrow‐derived macrophages. *WT* and *Gpr109a* ^−/−^ bone marrow chimeric mice were intragastric administrated with 3‐HB or without 3‐HB (100 mg kg^−1^ d^−1^) for 9 weeks. A,B) Representative images and quantitative analysis of lesion areas, Masson measurement of lesion areas (*n* = 7). C,D) Representative images and quantitative analysis of lipid deposition, respectively. Oil Red O staining measurement of lipid content (*n* = 5). Results were presented as mean±SEM from at least three independent experiments, Student's *t*‐test, * *p*< 0.05, ** *p* < 0.01.

To further verify that 3‐HB attenuates atherosclerosis in vivo via macrophages with high expression of Gpr109a, we generated two bone marrow chimeric mouse models by transplanting bone marrows from *WT* or *Gpr109a* ^−/−^ mice to lethal dose irradiation‐treated *apoE* ^−/−^ mice, respectively. Four weeks after transplantation, mice were fed with a high‐fat diet in the absence or presence of 3‐HB (100 mg kg^−1^ d^−1^) for 9 weeks. In *WT Gpr109a* ^−/−^ chimera mice treated with or without 3‐HB, it was found that 3‐HB significantly reduced the lesion areas and lipid deposits in the *WT apoE* ^−/−^ chimeric mice compared with that of its control (**Figure** **7**A,B). As expected, no difference was observed in the *Gpr109a* ^−/−^ *apoE* ^−/−^ bone marrow chimeric mice treated with or without 3‐HB (Figure 7C,D). These results suggested that the antiatherosclerotic effect of 3‐HB in *apoE* ^−/−^ mice depended on bone‐marrow‐derived cells expressing Gpr109a. As macrophages are the main components of atherosclerotic plaques, we can still conclude that 3‐HB mainly exerted its atheroprotection via the macrophages expressing Gpr109a.

## 3 Discussion

3‐HB is an endogenous molecule produced via fatty acid *β*‐oxidation during starvation or intense exercise. In plasma, the concentration of 3‐HB could reach (1–2) × 10^−3^ m fasting for 2 days and could reach (6–8) × 10^−3^ m after fasting for a prolonged time.^[^ ^45^ ^]^ 3‐HB can be easily and quickly metabolized in vivo that seems to limit its application.^[^ ^46^ ^]^ Ketogenic diet with a very low amount of carbohydrate mimicking the starvation mode of the body can consistently maintain the level of 3‐HB in the range of (2–8) × 10^−3^ m in animals.^[^ ^47^ ^]^ Ketogenic diet, which has been clinically used to treat refractory epilepsy in children,^[^ ^48^ ^]^ can ameliorate polycystic kidney disease^[^ ^49^ ^]^ and impact the composition of gut microbiota due to the production of 3‐HB.^[^ ^50^ ^]^ However, this dietary intervention is hard for people to adhere to it. In our previous work, we have demonstrated that orally administrated 3‐HB has a protective role of Alzheimer's disease, osteoblast, and microgravity‐induced osteoporosis.^[^ ^51^ ^]^ 3‐HB is available as a dietary supplement in the USA and, as demonstrated in this study, it could ameliorate features of atherosclerosis orally administrated only once a day even in the presence of a high‐fat diet. As atherosclerosis is a long‐term chronic disease related to diets,^[^ ^52^ ^]^ direct supplement of 3‐HB is more convenient and feasible than ketogenic diet. Compared with another Gpr109a agonist, the antiatherosclerotic drug niacin, which has been used clinically for more than 30 years, has limited its application due to its side effects such as skin flushing,^[^ ^17^, ^53^ ^]^ 3‐HB maybe a promising option for long‐term treatment of atherosclerosis.

This study reports that the exogenous administration of 200 mg kg^−1^ d^−1^ of 3‐HB resulted in potent fat reduction in *apoE* ^−/−^ mice even in the presence of a high‐fat diet. Interestingly, at a concentration of 200 mg kg^−1^ d^−1^, 3‐HB did not show a significant influence on the lipid profile and levels of inflammatory cytokines. It was reported that activation of the same GPCR using different concentrations of the same ligands or distinct ligands could induce diverse activation modes of GPCR leading to different responses.^[^ ^54^ ^]^ It is speculated that the 3‐HB concentration could account for this phenomenon observed. Since this study mainly focused on the mechanism of 3‐HB related to the chronic inflammatory response in atherosclerosis mice, we did not evaluate the role of 3‐HB at the concentration of 200 mg kg^−1^ d^−1^ at pathology level. 3‐HB at this concentration may be of great potential for attenuating atherosclerosis; it is highly likely other unknown mechanism relevant to its reduction on body weight, yet it could not be associated with chronic inflammation. For our next work, we will further optimize 3‐HB doses and explore the precise relationship between 3‐HB concentration and its cell cytophatic effect.

For its molecular mechanisms in atherosclerosis, we found that 3‐HB decreases the M1 inflammatory proportion and reduces cholesterol accumulation depending on its receptor Gpr109a in macrophages for the first time. We found that the protective effects of 3‐HB disappeared in *Gpr109a* ^−/−^ and *NLRP3* ^−/−^ BMDM cells, indicating that 3‐HB attenuates atherosclerosis via the Gpr109a–NLRP3 pathway. Ca^2+^ is a pivotal second messenger in cellular signaling transduction.^[^ ^55^ ^]^ Here, we demonstrated that 3‐HB–Gpr109a signaling‐mediated Ca^2+^ influx reduced the Ca^2+^ release from ER, thereby maintaining ER Ca^2+^ storage homeostasis, and inhibited ER Ca^2+^ storage depletion induced NLRP3 inflammasome activation. NLRP3 inflammasome is a key link between innate inflammatory response and cholesterol metabolism.^[^ ^34^ ^]^ Our research provides direct evidence on how 3‐HB affects this immune‐metabolic cross talk. This modulation of 3‐HB and its receptor Gpr109a allow the exploitation of new therapies to manage other complex NLRP3‐driven immune‐metabolic diseases.

These data also demonstrate that there is a new connection between GPCRs’ signaling‐induced Ca^2+^ influx and the NLRP3 inflammasome activation. Our results, for the first time, show that 3‐HB–Gpr109a signaling promotes the influx of Ca^2+^ under a normal physiological state and 3‐HB–Gpr109a signaling‐mediated Ca^2+^ influx represses NLRP3 inflammasome activation through PLC‐IP3R pathway. Substantially increasing extracellular Ca^2+^ was revealed to activate the NLRP3 inflammasome through GPRC6A via the PLC–IP3R cell signaling pathway.^[^ ^24^, ^25^ ^]^ These observations indicate that excessive extracellular Ca^2+^ triggers calcium influx through GPCRs, and the activation of GPCRs results in Ca^2+^ influx; they have totally reverse effect in the activation of NLRP3 inflammasome. Therefore, further research is needed to investigate the relationship between the quantitative concentration of influx of extracellular Ca^2+^ and the activation of NLRP3 inflammasome.

In summary, for the first time, we demonstrate that daily oral administration of 3‐HB once can attenuate atherosclerosis in *apoE* ^−/−^ mice. The mechanism can be attributed to the capacity of 3‐HB to restore homeostasis of innate immune response and cholesterol metabolism in atherosclerosis. 3‐HB suppresses the inflammation and reduces the cholesterol retention through the Gpr109a–NLRP3 pathway by promoting the influx of extracellular Ca^2+^ in macrophages. Our studies have provided preclinical evidence that 3‐HB could be developed into an effective antiatherosclerosis therapy for future clinical usages.

## 4 Experimental Section

##### Experimental Model Animal

*ApoE* ^−/−^ mice were purchased from Beijing Vital River Laboratory Animal Technology Co.; *Gpr109a* ^−/−^ mice were kindly donated by Prof. Wei Wang of Jilin University, while *NLRP3* ^−/−^ mice were purchased from Dr. Yong‐gang Lu of Hebei General Hospital Clinical Laboratory.

All mice were housed in a specific pathogen‐free animal facility in Tsinghua animal house, with a 12 h light and 12 h dark cycle. All mice were fed a high‐fat diet containing 1.25% cholesterol (D12108C, Research Diets, Inc.). Mice were randomly divided into five groups (*n* = 7–9 per group) for daily intragastric administration for 9 weeks: negative control, an equal volume of phosphate buffer saline (PBS); 3‐HB (298360, Sigma) (50, 100, and 200 mg kg^−1^ d^−1^), and niacin (170860, Sigma) (100 mg kg^−1^ d^−1^, positive control) were used for administration daily, respectively. All animals were weighted every week. After 10 weeks of atherogenic diet consumption, mice were fasted for 12 h and then were sacrificed. The blood samples were collected, and then the mice were perfused in situ with cold PBS through the left ventricle. After that, hearts and aortas were dissected, and some of the tissues were fixed in 4% paraformaldehyde for histopathological analysis, and some aortas were used for protein extractions. All procedures were reviewed and approved by the Institutional Animal Care and Use Ethic Committee at Tsinghua University (NO. 15‐CGQ3).

##### Plasma Lipid and Lipoprotein Biochemical Analysis

After 12 h of fasting, mice were anesthetized, and blood samples were collected via cardiac puncture. Serum lipid profiles, including total cholesterol (TC), total triglyceride (TG), low‐density lipoprotein cholesterol (LDL‐C), and high‐density lipoprotein cholesterol (HDL‐C) were measured by enzymatic assay with an automatic biochemical analyzer (Mindray, BS‐350E), respectively.

##### Cytokines Assay

Serum was assayed using commercial enzyme‐linked immunosorbent assay (ELISA) kits for murine IL‐6 (1210602), TNF‐*α*(430907), and IL‐1*β* (1210122) (Biolegend) according to the manufacturer's instructions.

##### Quantification of Atherosclerosis

Whole aortas were dissected, and open aortas were fixed en face and then stained with 0.5% Oil Red O (O0625, Sigma) propylene glycol solution for 30 min to detect aortic plaque burden. The digital images were taken and then were analyzed by Image‐Pro Plus 6.0 software.

The hearts were embedded in optimal cutting temperature (O.C.T.) compound (4583, Sakura) and frozen at −80 °C. Frozen tissues were layer‐by‐layer sectioned with a thickness of 8 m from the aortic sinus using the cryo‐cut microtome (Lecia, CM1950).

The aortic sinus sections were fixed, and Movat staining was performed for the analysis of lesion areas. The macrophages inside the atheromatous plaques were analyzed using immuno‐histochemistry for CD68. CD68^+^ areas were analyzed by Image‐Pro Plus 6.0 software. Aortic sinus sections were stained with 0.5% Oil Red O propylene glycol solution for lipid deposition in the plaques, and the Oil Red O stained area was quantitatively analyzed using the Image‐Pro Plus 6.0 software.

##### Flow Cytometric Analysis of Macrophage Differentiation

For *in vivo* analysis, *apoE* ^−/−^ mice treated with or without 3‐HB were anaesthetized with isoflurane (26675‐46‐7, J&K). The spleens were dissected, and grinded cells were filtered through a 100 µm cell strainer (352360, BD), and red blood cells were lysed with the ammonium chloride potassium (ACK) lysing buffer (A1049201, Gibco) on ice. Cells from the spleens of *apoE* ^−/−^ mice were incubated with labeled antibodies at room temperature for 30 min as follows: fluorescein isothiocyanate (FITC)‐CD45 (103108), peridnine chlorophyll protein/cyanine (Percp/cy) 5.5‐F4/80 (123128), preeclampsia (PE)‐CD11b (101207), allophycocyanin/cyanine (APC/cy) 7‐CD11c (117323), and preeclampsia/cyanine (PE/cy) 7‐CD206 (141720) according to the manufacturers recommended procedures, followed by washing three times with PBS and then analyzed by flow cytometry (BD FACS Aria III, USA). All the antibodies were purchased from BioLegend.

For *in vitro* analysis of macrophage differentiation, BMDM cells were incubated with LPS (100 × 10^−9^ g mL^−1^), and co‐treated with or without 3‐HB (10 × 10^−3^ m) for 24 h. Then the cells were collected and were incubated with labeled antibodies at room temperature for 30 min as follows: Percp/cy5.5‐F4/80 (123128), PE‐CD11b (101207), APC/cy7‐CD11c (117323), and PE/cy7‐CD206 (141720) according to the manufacturer's instructions, Subsequently, BMDM cells were washed three times with PBS and then analyzed by BD FACS Aria III. All the antibodies were purchased from BioLegend.

##### Immunofluorescence Staining

Immunofluorescence staining was performed as reported,^[^ ^56^ ^]^ and fixed sections were blocked with 5% bovine serum albumin (BSA) and then incubated with primary antibodies overnight at 4 °C, followed by incubation with secondary antibodies conjugated with fluorescent dyes at 37 °C for 1 h. For the analysis of M1 macrophage proportion, co‐localization of M1 macrophage marker iNOS and macrophage marker CD68 were evaluated using immunofluorescence staining. Anti‐iNOS antibody (ab178945) and anti‐CD68 antibody (ab53444) were purchased from Abcam. For studying the effect of 3‐HB on cholesterol efflux in macrophages, cells were incubated using anti‐ABCA1 antibody (NB400‐105), and for co‐localization staining, anti‐CD68 antibody (ab53444) was used. Images were captured by a fluorescence microscope (Andor, Dragonfly, England) and were quantitatively analyzed using Image‐Pro Plus 6.0 software.

##### Bone Marrow Transplantation

Bone marrow transplantation (BMT) was conducted as reported.^[^ ^14^, ^57^ ^]^ Briefly, recipient mice (male *apoE* ^−/−^ mice, 6 week old) were fasted overnight, then irradiated (10 Gy, 5 Gy twice with a 2 h interval), and then transplanted with bone marrow cells. Bone marrow cells were obtained aseptically from tibiae and femora of *WT* and *Gpr109a* ^−/−^ mice, respectively. These cells were resuspended in sterile PBS and transplanted by intravenous infusion into lethally irradiated *apoE* ^−/^ *^−^* recipients 1 day after irradiation. Four weeks after BMT, mice were fed an atherogenic diet containing 1.25% (w/w) cholesterol for 9 weeks.

##### Isolation and Culture of Bone BMDM Cells

Bone‐marrow‐derived cells were isolated from femur and tibia bones of 6 week old mice. Red blood cells were removed using the ACK lysing buffer (A1049201, Gibco), then centrifuged and re‐cultured in the BMDM growth medium (high‐glucose Dulbecco's modified Eagle's medium (DMEM, Gibco) with 20% L929 cell supernatant medium,10% fetal bovine serum (FBS, Gibco), 10010^−6^ g mL^−1^ penicillin and streptomycin), and then changed with the fresh BMDM growth medium on the third day. After 7 days of culture, cells were harvested and used for the following experiments.

##### Cholesterol Efflux Assay

Experiments were performed as reported.^[^ ^16^ ^]^ 5 × 10^5^ BMDM cells per well were plated on µ‐slide 8 well (Thermo Fisher) with DMEM containing 10% FBS overnight. Thereafter, BMDM cells treated with or without 3‐HB (10 × 10^−3^ m) were loaded with NBD‐cholesterol (N1148, Invitrogen) in DMEM containing 2.5% FBS for 1 h at 37 °C. Subsequently, these cells were washed twice with PBS and placed in serum‐free medium supplemented with 25 × 10^−6^ mL^−1^ high‐density lipoprotein 3 (HDL3, L1557, Sigma) for 1 h, then these cells were washed twice and stained with 4′,6‐diamidino‐2‐phenylindole (DAPI), followed by fixing and recording the fluorescence intensity using confocal microscopy (Andor, Dragonfly, England).

##### Measurement of Cytosolic Calcium Concentration

BMDM cells from *WT* mice and *Gpr109a* ^−/−^ mice were plated on glass‐bottom chambers (NEST) overnight, respectively, and were incubated with Fura‐2 AM (10 × 10^−6^ m) at 37 °C for 30 min, and then perfused with different concentrations of 3‐HB (1 × 10^−3^, 5 × 10^−3^, and 10 × 10^−3^ m) and its positive control niacin (0.1 × 10^−3^ m), respectively, in Hank's buffer with or without added calcium ions at a flow rate of 1 mL min^−1^. Fluorescent Ca^2+^ signal was studied using a Ca^2+^ detection system (Photon Technology International, Inc. or PTI, Edison, NJ, USA). Fura‐2 AM was excited alternately at 340 and 380 nm (with monochromater DeltaRam X), and emitted fluorescent images were collected with a CCD (NEO‐5.5‐CL‐3, Andor/Oxford Instruments, UK) at 510 ± 25 nm. The fluorescent ratios of F340/F380 were plotted against time with Sigmaplot10 as reported previously.^[^ ^58^ ^]^

BMDM cells from *WT* mice were plated on glass‐bottom chambers (NEST) overnight, respectively, and were incubated with Fluo‐4 AM (10 × 10^−6^ m) at 37 °C for 30 min, followed by perfusion with Hank's buffer without adding Ca^2+^ at a flow rate of 1 mL min^−1^. Fluorescent Ca^2+^ signal was studied using a fluorescence microscope (Andor, Dragonfly, England). Fluo‐4 AM was excited alternately at 488 nm and emitted fluorescent images at 510±25 nm. The fluorescent intensity was plotted against time with Sigmaplot10.

##### Western Blot Analysis

Proteins were purified from treated BMDMs or the aortas of mice. Total protein was harvested using radio immunoprecipitation assay (RIPA), cell lysis buffer (Beyotime) containing protease inhibitors, and phosphatase inhibitors (A32959, Pierce). Protein concentration was quantified using a bicinchoninic acid (BCA) assay kit (Beyotime) according to the manufacturer's instruction. For analysis of western blot, proteins were separated on sodium dodecyl sulfate polyacrylamide gel eletrophoresis (SDS‐PAGE) gels and transferred to polyvinylidene fluoride (PVDF) membranes (Millipore). The membranes were blocked in 5% BSA buffer, followed by incubation with primary antibodies for 24 h overnight. After washing and incubation with the horseradish peroxidase (HRP)‐labeled secondary antibody, an enhanced chemiluminescence (ECL) substrate‘s detection (Engreen) was used to detect protein expression, and the signals were captured using an imaging system (Bio‐Rad, ChemiDoc XRS+, USA). The gray value of protein bands was analyzed using the Image J software. Primary antibodies were used as follows: ABCA1 (NB400‐132) and ABCG1 (NB400‐105) antibodies were purchased from Novus Biological; iNOS (Cat# 13120), IL‐6 (.Cat# 12912), PLC (Cat# 3356), p‐PLC (Cat# 14247), p‐IP3R (Cat# 8548), IP3R (Cat# 8568), p‐CaMKII (Cat# 3356), CaMKII (Cat# 50049), PERK (Cat# 5683), p‐PERK (Cat# 3179), ATF6 (Cat# 65880), and IL‐1*β* (Cat# 31202) antibodies were purchased from Cell Signaling Technology. IL‐18 (ab71495), p‐IRE1*α*(ab48187), IRE1*α*(ab37073), and glyceraldehyde‐3‐phosphate dehydrogenase (GAPDH) (ab181602) antibodies were purchased from Abcam Company. HRP‐labeled secondary antibody was purchased from Cell Signaling Technology.

##### Statistical Analysis

Results were presented as mean±standard error of mean (SEM). The sample size (*n*) for each experiment was included in the relevant figure legends. The statistical differences between two groups were analyzed using the unpaired two‐tailed Student's *t*‐test, and comparisons among three or more groups were performed using one‐way analysis of variance (ANOVA) by GraphPad Prism 6.0 software. The data were considered significant difference as values of *p* < 0.05, * *p* < 0.05, ** *p* < 0.01, *** *p* < 0.001, and **** *p* < 0.0001.

## Conflict of Interest

The authors declare no conflict of interest.

## Author Contributions

G.‐Q.C. supervised this study; Y.‐d.Z., J.C., and F.‐q.W. conducted parts of the experiments; S.‐j.Z. and Z.‐h.L. performed most studies and analyzed results, respectively. W.W. constructed the *Gpr109a* ^−/−^ mice. Y.L. and Z.‐J.C. studied the Ca^2+^ signaling.

## Supporting information

Supporting Information

## Data Availability

The data that support the findings of this study are available from the corresponding author upon request.
