# Supplementary material for: Ketone Body 3‐Hydroxybutyrate Ameliorates Atherosclerosis via Receptor Gpr109a‐Mediated Calcium Influx
Source: Adv Sci (Weinh). 2021 Mar 1;8(9):2003410. doi: 10.1002/advs.202003410 (PMC8097358; doi:10.1002/advs.202003410)
Supplement: Supplementary file 1 — Supporting Information [file ADVS-8-2003410-s001.pdf]

© 2021 Wiley-VCH GmbH

ADVANCED SCIENCE
Open Access

Supporting Information

for *Adv. Sci.*, DOI: 10.1002/advs.202003410

Ketone Body 3-Hydroxybutyrate (3-HB) Ameliorates Atherosclerosis via Receptor Gpr109a Mediated Calcium Influx

*Shu-jie Zhang, Zi-hua Li, Yu-dian Zhang, Jin Chen, Yuan Li, Fu-qing Wu, Wei Wang, Zong-Jie Cui, Guo-Qiang Chen**

Supporting Information

Title: Ketone Body 3-Hydroxybutyrate (3-HB) Ameliorates Atherosclerosis via Receptor Gpr109a Mediated Calcium Influx

*Shu-jie Zhang, Zi-hua Li, Yu-dian Zhang, Jin Chen, Yuan Li, Fu-qing Wu, Wei Wang, Zong-Jie Cui, Guo-Qiang Chen**

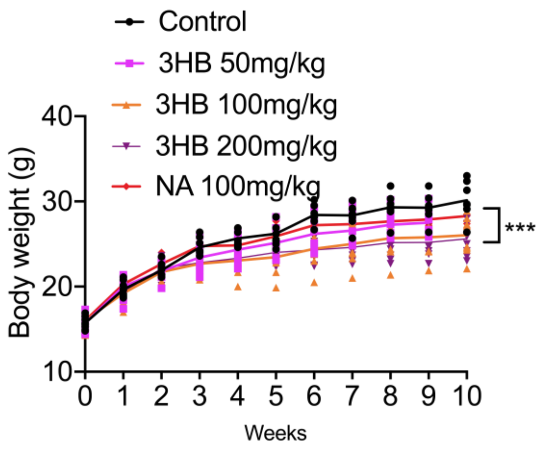

Figure S1. Changes of body weights resulting from different treatments of 3-HB in *apoE*$^{-/-}$ mice fed with a high fat diet for 10 weeks (n=7-9). Results were presented as mean ±SEM from at least three independent experiments, one-way ANOVA, *** $p < 0.001$.

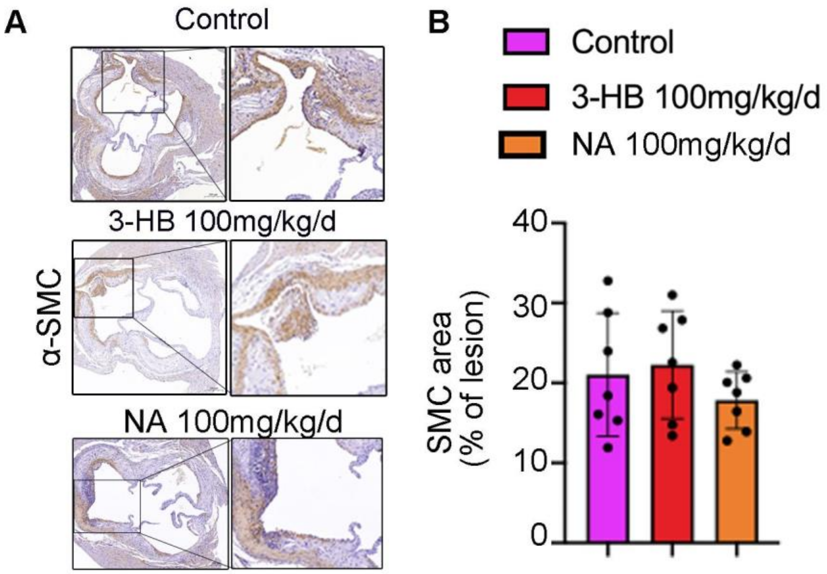

Figure S2 A) Representative images of smooth muscle cells areas in aortic root sections of mice treated with or without 3-HB and NA, α-SMC immunohistochemical staining for smooth muscle cells. B) Quantification analysis of smooth muscle cells areas (n=7), Scale=200 μm. Results were presented as mean±SEM from at least three independent experiments, one-way ANOVA, no statistical significance was found.

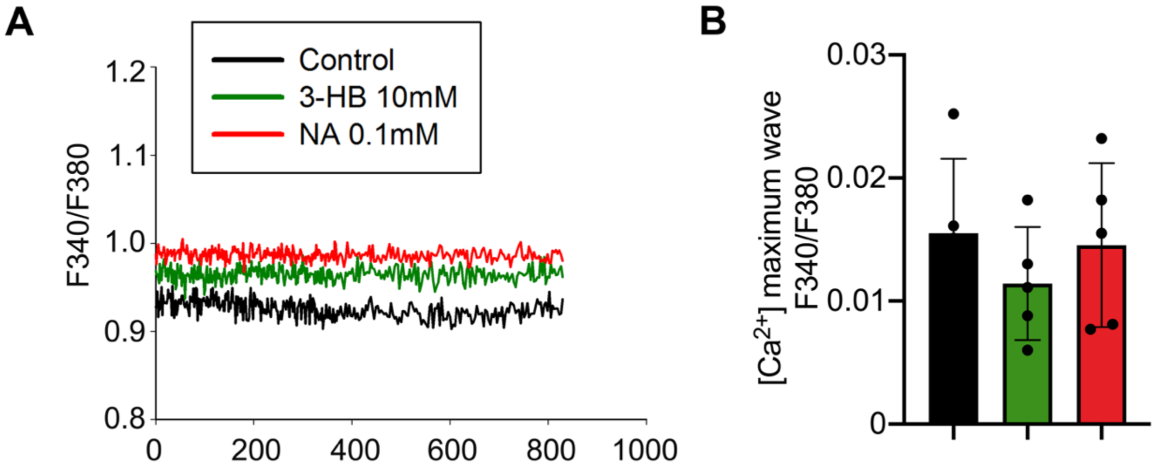

Figure S3 A) Intracellular Ca^{2+} concentration in real time detected by Fura-2 fluorescence imaging. Isolated BMDMs from *WT* mice were loaded with Fura-2 AM (10×10^{-6} M) and perfused in Hank's buffer

without Ca^{2+} addition. 3-HB (10×10^{-3} M) or NA (0.1×10^{-3} M) were added as indicated by the vertical line. (B) Net increases in intracellular Ca^{2+} concentration were calculated and plotted as shown (n=5). Results were presented as mean±SEM from at least three independent experiments, one-way ANOVA, no statistical significance was found ($p > 0.05$).
